# *The need for adjusting the script*; characteristics and future directions of deployable healthcare facilities for climate-change-induced disasters

**DOI:** 10.3389/fpubh.2026.1872329

**Published:** 2026-09-04

**Authors:** Noemi Bitterman, Michele Di Marco, Alberto Martinetti

**Affiliations:** 1 Faculty of Architecture and Town Planning, Technion Institute of Technology, Haifa, Israel; 2 Department of Architecture and Design (DAD), Politecnico di Torino, Turin, Italy; 3 Humanitarian Engineering Research Group, Faculty of Engineering Technology, University of Twente, Enschede, Netherlands

**Keywords:** climate change, continuity of care, deployable healthcare facilities, disasters, emergency design, extreme environment

## Abstract

Climate change increasingly disrupts public health through frequent, severe disasters, rendering traditional deployable hospitals inadequate for unpredictable climate demands. This paper examines the unique requirements for healthcare infrastructure in extreme environments and proposes a shift toward advanced technological solutions, including air-inflated structures, smart materials, 3D printing, and autonomous robotics. We advocate for modular, sustainable, and age-friendly designs that integrate mental health and community services and remain in the field beyond the acute disaster phase, to ensure continuity of care. Realizing this vision requires a multidisciplinary task force of architects, engineers, and healthcare professionals working together to build resilient, adaptive infrastructure for an escalating climate crisis.

## Introduction

The World Health Organization defines climate change as the greatest challenge to health in the current century, as it affects all aspects of human health at the individual and population level (1), and therefore calls upon healthcare systems to prepare accordingly (2). While the media increases its engagement with climate change and the number of publications on the effects of climate change on health, society, and the economy grows (3, 4), less studies are available on healthcare facilities for providing an adequate and immediate response in disasters caused by the climate crisis (2).

Several reviews address mobile hospitals for natural disaster situations (5–8), while some focus on specific emergency responses to disasters, such as earthquakes (9), storms (10), or floods (11). Studies regarding deployable healthcare facilities for climate-change-induced disasters are, however, still lacking. This article examines the impacts of climate-change-induced disasters on public health and the implications on planning deployable hospitals for such situations.

**Figure 1:**
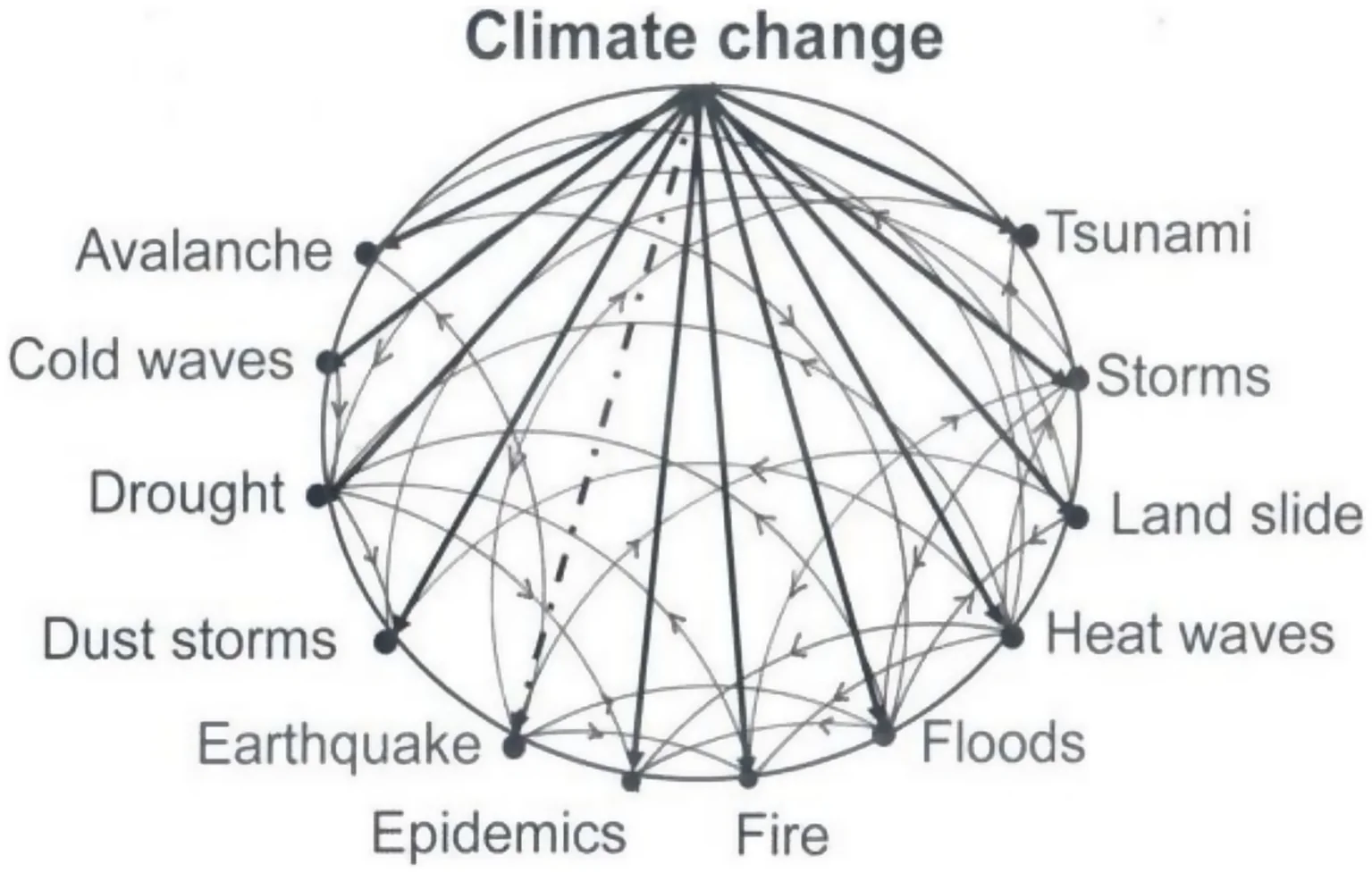
Climate change and natural disasters (listed in alphabetical order), with a schematic outline of possible interactions. Straight lines represent direct effects, while curved lines indicate interactions and cascading disasters.

Climate change triggers *climatological* disasters such as hot and cold waves, drought and wildfire; *meteorological* events of storms; and *hydrological* hazards including floods, avalanches, and landslides (12, 13) (Figure 1). Partial evidence links climate change to geophysical disasters such as earthquakes (12) (dashed line in Figure 1). Interrelations between natural disasters occur frequently (Figure 1 curved lines), often resulting in synergistic and cascading events. These interactions and spill overs further complicate environmental conditions, economy, society, healthcare needs and the function of deployable healthcare facilities (2).

## Clinical manifestations of climate change-induced disasters

*Extreme temperatures* (hyperthermia) cause dehydration and heat stroke, which threaten the functioning of the heart and nervous system (2–4, 14). High temperatures may also trigger pandemics involving viral, bacterial, and parasitic infectious diseases, as well as insect infestations (4, 12, 15). *Floods*, a most common natural hazard caused by climate change, may lead to extensive morbidity and mortality (16–18). The immediate health impacts of floods include drowning and near-drowning, injuries, hypothermia, animal bites, injuries from debris, chemical contamination, upper respiratory tract infections, and diarrhoea (16, 18, 19). Further consequences of floods encompass infectious diseases, malnutrition, poverty-related diseases, and diseases associated with population displacement. Floods can potentially increase the transmission of communicable diseases, including water-borne diseases (typhoid fever, cholera, leptospirosis, and hepatitis A) and vector-borne diseases (malaria, dengue and dengue haemorrhagic fever, yellow fever, West Nile Fever (15)). *Storms* such as tornadoes or tropical storms (e.g., hurricanes, cyclones) cause head trauma and emergency surgeries, orthopaedic injuries, respiratory diseases, allergies due to dust, especially for people with asthma and pre-existing diseases, diarrhoea, and increased incidence of animal and insect bites (17, 19). *Earthquakes* result in emergency surgeries, head trauma, and orthopaedic injuries (17), thus requiring surgical assistance, especially for limb injuries. *Wildfires* result in smoke, burns, respiratory problems, and exacerbate chronic obstructive pulmonary diseases (COPD) and asthma (17, 19).

## Indirect effects and spillover of climate change-induced disasters

*Environmental consequences* associated with climate change, such as water contamination, air pollution, electricity disruption, poor sanitation, food shortage, transportation disruption, and ecological changes, profoundly affect health (2, 4, 19). *Mental health vulnerabilities* often accompany disasters and emergencies, including stress, anxiety, instability, depression, worry, fear, grief, anger, and suicidal thoughts (2). These factors highlight the urgent need for immediate mental health support in deployable healthcare facilities (2, 3, 8). A special attention should be given to vulnerable populations such as children and older adults (2–4, 14), with protocols specifying ongoing therapy to prevent post-traumatic stress disorder (PTSD). Survivors commonly experience *decreased physical activity*, due to challenging environmental conditions such as extreme heat or cold, high humidity, polluted air, physical damage to infrastructure, flooding, and uneven terrain that restrict mobility. These limitations contribute to increased rates of obesity and cardiovascular disease in the affected population (4, 14). *Nutrition insufficiency and malnutrition* occur in most disaster situations, either directly or as a consequence of intestinal diseases from infections, vector-borne diseases, water contamination, infected and poisoned food, reduced quality and availability of fresh vegetables and fruit, and sanitation problems (16, 19). *Low adherence to medical treatment and medication intake* is common in disaster areas, due to the destruction of houses and inaccessibility of essential medical homecare devices such as respiratory equipment, glucometers, blood pressure monitors, and disruption of home medical services of family physician or nurses’ visits (2). The evacuation of patients from nonfunctioning hospitals in disaster areas, the collapse of healthcare infrastructure, shortages of medical personnel, lack of outpatient ambulatory services, and the inability to supply treatments such as dialysis and cancer treatments, combine to worsen survivors’ conditions, placing an increased burden on temporary hospitals deployed at the disaster area.

## Characteristics and needs of healthcare facilities in climate-change-induced disasters

Growing variability and unpredictability in climate conditions require planners to adopt a versatile and dynamic holistic approach in designing deployable healthcare facilities. Studies have shown that the disruption of medical services is the most significant health damage in natural disasters (8). Therefore, it is essential that deployable hospitals should be integrated with existing community healthcare services and social services to address climate-change-induced disasters effectively (8, 20). Deployable hospitals should have the capacity to serve as both independent healthcare facilities but also as supplementary facilities for existing health facilities that were damaged, destroyed or non-functioning due to the lack of sufficient personnel, equipment, and/or energy sources (10). Deployable hospitals must provide immediate support and ambulatory services such as chemotherapy and dialysis to patients who cannot access standard hospital care. Therefore, emergency staff should include community doctors, who bring basic surgical skills and can treat communicable and non-communicable diseases, (8), nurses with wound care expertise, and physiotherapists and orthopaedic technicians, to deliver early rehabilitation to affected populations.

Deployable healthcare facilities must integrate mobile technology (mHealth) and information and communications technology (ICT) into their work protocols from the initial stages of construction in the disaster area (21). mHealth allow faster processing and transport of patients, improved accuracy of triage, better monitoring of patients, direct access to medical records and central health services, thus creating order in chaotic disaster situations and improving the continuity of care (21).

Deployable hospitals in natural disaster areas encounter unpredictable and fluctuating weather and environmental conditions, such as rain, winds, landfall, and extreme temperatures typical of climate change. Therefore, deployable hospitals should be flexible, modular, and able to withstand variable external conditions. Flexibility enables multifunctional, adaptable spaces, allowing hospitals to respond to changing environmental conditions and different needs of patients throughout their operational lifespan (8, 22). Healthcare facilities that are built from modular units can be quickly reconfigured for various sizes and are easy to transport (7). Flexible facilities last longer, serve multiple purposes, accommodate changeable users’ needs more effectively, offer economic and ecological advantages, and remain relevant to cultural and societal trends even after the acute phase of disaster has passed (22, 23).

Deployable facilities require both vertical and horizontal transportation systems for patients and supplies to overcome damaged or inaccessible roads, including autonomous systems such as drones, ground robots, unmanned surface vehicles (USVs), and hovercraft (8, 24). Additionally, the flooring system of the healthcare facility should be designed to elevate autonomously, ensuring safe entry and movement between wards in conditions such as flooding, heavy rain, unstable surface or muddy terrain.

Given the increase of aging population and the heightened vulnerability of older adults in disaster scenarios, developing age-friendly design for deployable healthcare facilities is crucial (2, 19). Age- friendly designs prioritize improved visibility, accessibility, multimodal signalling, and clear iconography (25). Understanding the specific needs of older adults and identifying their unique risk factors are essential strategies for enhancing both healthcare facility design and community resilience (2, 25).

## Technological solutions for healthcare facilities in climate-change-induced disasters

Deployable healthcare facilities must adhere to general healthcare standards and regulations, including requirements for medical gases, equipment, infection control, safety, and sterility (26). They must also meet specific criteria for deployability, such as mobility, portability, modularity, and flexibility (5, 7, 8). Furthermore, the climate crisis imposes additional challenges, requiring healthcare facilities to withstand extreme environmental conditions and adapt to unpredictable, rapidly changing disaster scenarios, including the cascading effects of multiple disasters (2, 3). Therefore, we suggest technological solutions, such as Airtecture, the use of smart materials, innovative building methods, autonomous systems and finally Biomimicry for source of inspiration on how Nature deal with natural disasters and extreme climate changes.

### A. Airtecture and smart materials

**Figure 2:**
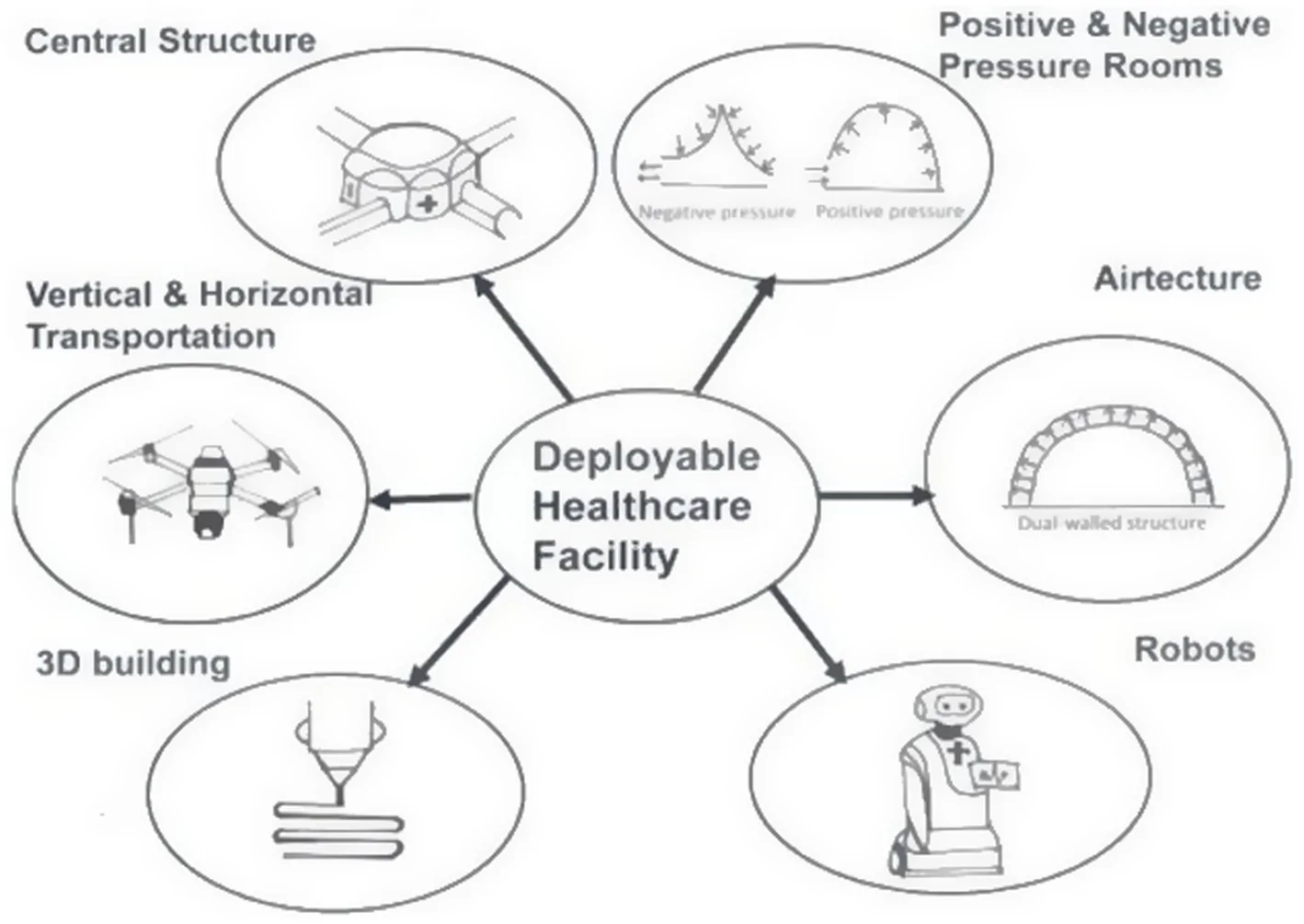
Scheme of some technological solutions for the structure and function of deployable healthcare facilities for climate crisis situations.

Air-inflated structures are already used for temporary public facilities such as sports arenas, concert halls, exhibition spaces, and entertainment venues. These structures are erected quickly for specific events for short term periods (5, 23, 27). Pneumatic structures are well-suited for deployable medical facilities because they can be transported in a compact form, inflated and deflated rapidly on-site, stored compactly for reuse, or repurposed for new functions in post-disaster situations (27). Air is a natural, sustainable, effective for thermal insulation and cost-free substance available everywhere and therefore ideal for changeable extreme temperatures everywhere and also in low- and middle-income countries. The modularity allowed through partial inflation, based on the number and type of patients and the unique design of the frame system ensures the entire structure remains stable even if a single unit is damaged. The military sector has adopted inflatable facilities due to their exceptional resistance to wear and tear. These structures may be constructed from strong, durable fabrics such as rubber-coated or PU-coated waterproof nylon which withstand harsh environmental conditions and extreme temperatures (28). They can also utilize flame-resistant fabrics like Kevlar, heat-resistant synthetic fibers, or bulletproof composites (29) effective for cases in which natural disasters may escalate into violent conflict. Smart materials, flexible wiring, sensors, actuators, climate-control tubing, communication cables, LED lighting units, flat solar panels, and even antibacterial components can be integrated seamlessly into the flexible walls. The internal pressure of the facility can be automatically adjusted to changing environmental conditions such as wind, rain, high temperatures or snow via a telemetric system that monitors the environment continuously. Additionally, the pneumatic system can rapidly create isolation rooms with positive and negative pressure (Figure 2), tailored to specific clinical needs that are essential for infectious disease control in climate-related disasters.

Dome-shaped pneumatic structures are aerodynamic and can withstand strong winds (23, 29). A central round tent with several inflated tunnel units for entry and exit provides convenient, multidirectional access to the hospital (Figure 2). Transparent panels integrated into the flexible walls may fill the interior with natural light, strengthening the connection with the external environment and enhancing the occupants’ sense of time and place.

### B. Innovative building methods *in situ*: 3D building

Innovative building methods using 3-dimensional (3D) printing technologies can enable the rapid and safe construction of deployable hospitals on-site, in complex and hazardous environments, and can even utilize local debris and materials (Figure 2). 3D building helps overcome time constraints and transportation limitations without exposing the rescue staff to risk (30). 3D building allows the integration of solar panels on the structure, passive cooling or heating features that reduce reliance on external energy, embedded sensors, monitoring and communication elements directly into the structure. Moreover, 3D printing can be used to produce a wide range of items in the point of need, from furniture for the healthcare facility to orthopaedic, surgical and small equipment, helping to overcome supply chain disruption, equipment shortages, and the need for equipment tailored to the specific location and situation (31, 32).

### C. Autonomous mobile platforms

Autonomous systems accelerate healthcare facility construction and support the healthcare operations in extreme weather conditions and hazardous insecure terrains, while minimizing risks to human live in the unsecure setting. Autonomous mobile platforms can assist in transporting injured people and supplies, through extreme weather conditions and over damaged or impassable roads that hinder movement.

Robots can be deployed for drug delivery, patient care, remote monitoring of physiological signals, and assistance with virtual family visits, helping to overcome shortage of medical staff, inaccessible environment, and challenges in understanding and communicating with local population (33, 34) (Figure 2). Since climate- related threat span diverse environments, including floods, rising sea levels, rugged and impassable terrain, or mud, it is recommended to use amphibious robots which offer significant advantages in terms of maneuverability, efficiency, and reliability, as they do not require switching between different propulsion systems for varying surfaces (35).

### D. Biomimicry inspired technology

Nature offers valuable models of resilience. Over millions of years, organisms have evolved to withstand extreme environmental conditions, adaptations that can be translated into engineering and architectural solutions (biomimicry). Few examples include insect shells and exoskeletons that inspire structural design; spider webs, known for high tensile strength, that inform flexible, tear-resistant building materials; termite mounds, valued for passive thermal regulation; and desert insects capable of harvesting water from moisture and dew (AskNature, https://asknature.org/) (36).

We propose that observing nature can inspire innovative solutions to the challenges posed by climate-change-induced disasters, as suggested in the ancient proverb: “Go to the ant, you sluggard; consider its ways and be wise” (Proverbs 6:6–11).

## Discussion

While the full depth and complexity of climate change impact on human health are not yet fully understood, there is no doubt that it represents an imminent, global threat, and healthcare system must prepare with unprecedented speed. We contend that our approach to temporary medical solutions must evolve beyond current disaster response paradigms. The existing models comprised of civilian and military mobile hospitals and humanitarian organizations deployed during acute events are insufficient for the unique and long-term challenges posed by climate-driven disasters.

Our argument is that future deployable healthcare facilities must function autonomously yet should be deeply integrated into existing local services. The facilities should remain in the field after the acute phase of the disaster to ensure continuity of care, a factor of paramount importance for population recovery (8). Deployable healthcare facilities should be established quickly, attentive to the needs of the place and the residents, while utilizing technological resources that will facilitate construction and performance, improve safety, and upgrade connection to country hospitals. Permanent hospitals must also prepare for the climate crisis and make structural changes in order to be ready for extreme and unexpected climate events and to treat a patient profile that will be typical of the direct and indirect effects of natural disasters. Digital technologies, remote connectivity, and artificial intelligence for data monitoring and processing are essential component for functionality of mobile hospitals.

Construction techniques should include 3D building methods, smart and innovative materials and autonomous robotic systems that provide professional, multilingual service in any terrain or weather, mitigating manpower shortages and reducing human risk. An inexhaustible reservoir is the exploration of solutions from nature, including animals and plants, for dealing with extreme situations and gaining inspiration from them.

Consequently, the design of transportable hospitals must move beyond the camouflage and functional aesthetic of traditional military models. Field healthcare facilities for climate change induced disasters should function as flexible, age-friendly platforms that integrate seamlessly into local healthcare landscapes and culture, thereby enhancing community resilience and mitigating cascading disaster effects. Where public healthcare systems lack the capacity to provide such infrastructure, the private sector must be harnessed, whether through voluntary humanitarian efforts or a sense of moral and national obligation (2). Recently, there has been growing interest in underground protected spaces that can serve during disasters related to climate crisis, particularly extreme temperatures, fires, storms, and its indirect events (37) including need for necessary floodproofing measures (e.g., raised access points, sealed barriers, and automated drainage pumps) to ensure viability during unexpected hydrological hazards. These facilities should be designed for “dual-use” functionality: serving community needs during routine times and transitioning into climate-emergency hospitals during crises. From these underground hubs, autonomous or manned mobile units can be deployed to provide targeted relief at disaster sites. Such centres must be equipped with stockpiles of food, medical equipment, and essential resources to ensure long-term resilience.

We propose the formation of a multidisciplinary task force, since the climate crisis is a complex and multidisciplinary event, much more than a single natural disaster. The multidisciplinary teams will include architects, engineers, emergency medicine professionals, WHO experts, psychologists, military personnel, and climate change specialists to develop a strategic plan for healthcare facilities that will be supported by research on the effectiveness of these solutions (2, 6, 8, 12). Designers must evolve structures to address emerging physiological and psychological needs, a thoughtful design strategy and layouts to reduce stress and fear, supporting the mental wellbeing of both survivors and responders aligned with local architectural traditions and cultural contexts. A solution for mobile hospitals for disaster situations originating from the climate crisis is much more than a one-off solution to a critical event. The climate crisis is an event with economic, social and even political consequences, and therefore planning hospitals for these situations will help improve the resilience of the population.

## Data Availability

The original contributions presented in the study are included in the article/supplementary material, further inquiries can be directed to the corresponding author.
